# Non-Adherence in Patients on Peritoneal Dialysis: A Systematic Review

**DOI:** 10.1371/journal.pone.0089001

**Published:** 2014-02-25

**Authors:** Konstadina Griva, Alden Yuanhong Lai, Haikel Asyraf Lim, Zhenli Yu, Marjorie Wai Yin Foo, Stanton P. Newman

**Affiliations:** 1 Department of Psychology, National University of Singapore, Singapore; 2 Department of Renal Medicine, Khoo Teck Puat Hospital, Singapore; 3 Department of Nephrology, Peritoneal Dialysis Centre, Singapore General Hospital, Singapore; 4 Unit of Behavioural Medicine, University College London, London, United Kingdom; 5 Health Services Research Group, City University London, London, United Kingdom; University of Ottawa, Canada

## Abstract

**Background:**

It has been increasingly recognized that non-adherence is an important factor that determines the outcome of peritoneal dialysis (PD) therapy. There is therefore a need to establish the levels of non-adherence to different aspects of the PD regimen (dialysis procedures, medications, and dietary/fluid restrictions).

**Methods:**

A systematic review of peer-reviewed literature was performed in PubMed, PsycINFO and CINAHL databases using PRISMA guidelines in May 2013. Publications on non-adherence in PD were selected by two reviewers independently according to predefined inclusion and exclusion criteria. Relevant data on patient characteristics, measures, rates and factors associated with non-adherence were extracted. The quality of studies was also evaluated independently by two reviewers according to a revised version of the Effective Public Health Practice Project assessment tool.

**Results:**

The search retrieved 204 studies, of which a total of 25 studies met inclusion criteria. Reported rates of non-adherence varied across studies: 2.6–53% for dialysis exchanges, 3.9–85% for medication, and 14.4–67% for diet/fluid restrictions. Methodological differences in measurement and definition of non-adherence underlie the observed variation. Factors associated with non-adherence that showed a degree of consistency were mostly socio-demographical, such as age, employment status, ethnicity, sex, and time period on PD treatment.

**Conclusion:**

Non-adherence to different dimensions of the dialysis regimen appears to be prevalent in PD patients. There is a need for further, high-quality research to explore these factors in more detail, with the aim of informing intervention designs to facilitate adherence in this patient population.

## Background

While the majority of End-Stage Renal Disease (ESRD) patients undergo in-center maintenance hemodialysis (HD) in most settings, chronic peritoneal dialysis (PD) is the dominant home dialysis therapy utilized by 11% of the dialysis population worldwide [1]. PD offers patients the convenience of home-based care and continuous clearance, but requires a daily commitment and a high level of involvement by patient and/or carer with scrupulous attention to hygiene so as to avoid infection of the peritoneum.

With increasing numbers of ESRD patients in need of Renal Replacement Therapy (RRT) and the need to expand dialysis delivery in home settings away from overburdened hospital and tertiary care settings, there is renewed interest in outcomes in patients who are established on PD regimes. Adherence to treatment is of paramount importance as non-adherence has been shown to have major consequences including an increased risk of mortality and hospitalization in patients on HD (see [2] for a review) and in PD [3], [4], which in turn lead to increased costs and expenditure for patient care [5], [6], [7]. However, in contrast to research on adherence in HD patients [2], [8], [9], [10], [11], [12] and other patient populations, little is known about adherence to PD regimes.

Researchers often use the terms compliance and adherence interchangeably, although they have slightly different implications. Compliance, principally used extensively in older literature, has drawn criticism for its emphasis on medical authority and an implication for patients as passive recipients of care. In response, the term adherence was introduced to recognize patients' right to choose whether or not to follow advice, calling attention to the importance of patients' active participation in their treatment regimes. It is also important and increasingly recognized that a distinction needs to made between intentional and unintentional non-adherence [13]. Non-adherence is unintentional when it is not deliberate through patients' lack of understanding, forgetfulness or miscommunication with health care professionals [14]. Intentional non-adherence, on the other hand, is when patients actively choose not to follow treatment recommendations, such as when they choose to delay, alter or skip dosages of prescribed medication, or to forego dietary or fluid intake recommendations. In this review the term adherence will be adopted, defined as the extent to which a person's behavior (taking medication, following a diet and/or executing lifestyle changes) corresponds with agreed recommendations from a healthcare provider [15]. Where available, intentional and unintentional non-adherence behaviors will be explored.

Overall, adherence among patients with chronic conditions is disappointingly low with rates estimated at 24.8% [16]. Evidence in HD patients shows a similar problem with non-adherence being common [17], occurring in 22–74% of dialysis patients depending on the definition of adherence and the type of treatment [10]. A review has documented up to 74% of HD patients as non-adherent to fluid restrictions, 81.4% for diet restrictions, and 73% for medication non-adherence [2]. The PD regimen is no less complicated and time-consuming than HD. PD patients are required to adhere to a demanding dialysis regime that involves regular manual exchanges at least thrice daily (in the case of continuous ambulatory peritoneal dialysis; CAPD), or long overnight dialysis exchange (in the case of automated peritoneal dialysis; APD), as well as lifelong changes in lifestyle related to diet, intake of multiple medications, and safety and preventive measures. None of the existing systematic reviews on adherence in dialysis to date has distinguished between adherence in HD and PD. PD patients tend to be younger, have fewer comorbidities, and be newer to RRT across varying populations [18], [19], [20], [21]. As the profile of the HD and PD populations differs, the value of a review focused on adherence in PD is therefore accentuated. Individual studies on the other hand have produced mixed results, with some reported higher levels of adherence in PD vs. HD, while others indicated lowered rates of adherence in PD [22] or no differences between the two dialysis modalities [23].

Because of this lack of evidence specific to PD patients in previous reviews, we have undertaken and report here a systematic literature review in which we aimed to:

1. Summarize and synthesize the frequency of (non-) adherence to dialysis exchanges, medication and diet/fluid intake in the PD population;
2. Compare rates of (non-) adherence to dialysis exchanges, medication and diet/fluid intake between patients on different PD modalities, i.e. APD and CAPD;
3. Examine socio-demographic, clinical, and psychological factors associated with adherence to dialysis exchanges, medication and diet/fluid intake.

## Methods

This systematic review follows the Preferred Reporting Items for Systematic Reviews and Meta-analyses (PRISMA) guidelines [24].

### Search strategy

Articles were identified through PubMed, PsychInfo, and CINAHL electronic databases using combinations of Medical Subject Heading (MeSH; where appropriate) terms and keywords: peritoneal dialysis; adheren*; complian*; medication*; diet*; fluid*; regimen*; session*; schedule*. Search results were downloaded and imported directly into EndNote X6, after which their bibliographic reference lists were scanned to identify additional relevant studies. The search was carried out in May 2013. Refer to Table 1 for an example of the search strategy for PubMed.

**Table 1:** Example Search Strategy (PubMed on 20/05/2013).

ID	Search
#1	Peritoneal Dialysis [MeSH Terms]
#2	Peritoneal Dialysis, Continuous Ambulatory [MeSH Terms]
#3	#1 OR #2
#4	Medication Adherence [MeSH Terms]
#5	Patient Compliance [MeSH Terms]
#6	#4 OR #5
#7	Medication*
#8	Diet
#9	Diets
#10	Dietary
#11	#8 OR #9 OR #10
#12	Fluid*
#13	#7 OR #11 OR #12
#14	Regimen*
#15	Session*
#16	Schedule*
#17	Exchange*
#18	#14 OR #15 OR #16 OR #17
#19	#13 OR #18
#20	#3 AND #6 AND #19

Note: Search string for PsycINFO and CINAHL was “(peritoneal AND dialysis) AND ((((((adheren*) OR nonadheren*) OR non-adheren*) OR complian*) OR noncomplian*) OR non-complian*) AND (((((((medication*) OR diet*) OR fluid*) OR regimen*) OR session*) OR schedule*) OR exchange*)”.

### Study selection

We used a three-step process to select the studies. First, using EndNote, duplicate articles were eliminated. Second, to discard irrelevant studies, two authors (HL and KG) screened all titles and abstracts of the papers. Disagreements between the authors were resolved by a consensus. The full paper was obtained where there was insufficient information in the abstract or title to determine eligibility. Third, to select studies that met our inclusion criteria, one analyst (HL) read the full papers identified at abstract screen. If the results of a study were reported in more than one publication, only the publication with the most complete results was retained. Only if publications on the same study focused on different outcomes (i.e. adherence to different aspects of treatment) or different populations were they included in this review.

Publications were included in this review only if full papers met the following criteria:

- written in English.
- published in peer-reviewed journals.
- included measure(s) of (non)-adherence outcome in either dialysis exchanges, medication, diet/fluid restrictions or exercise.
- explicated criteria/methods or cut offs to calculate and define (non-) adherence.

The papers were required to include details of the methods used to determine non-adherence in any one of the treatment aspects (i.e. dialysis exchanges, medication, diet/fluid restrictions, or exercise) and some numeric results on rates of non-adherence. As there is no gold standard adherence measure, all measures were considered (e.g., self-report, physician/nurse estimate, tablet count, and prescription refill, electronic monitoring, inventory checks/delivery records, built-in software or electronic monitoring systems) as long as the criteria for definition of non-adherence based on these measures and data on frequencies were reported. All definitions of (non-) adherence, such as the percentage of doses taken/exchanges performed over a given time period and percentage of patients achieving a specified adherence level or clinical target, were considered. Where multiple measures were reported, the percentage of patients achieving a specified adherence level was used in this review as this was common to the majority of studies.

Cohort studies, both prospective and retrospective, and cross-sectional designs were all included. For intervention studies, they were only considered if baseline rates were reported. Dissertations, systematic reviews, meta-analyses, case series, editorials, opinion papers, and interventions without any baseline rates on non-adherence were excluded. Studies were also excluded if they did not examine (non-) adherence on either performance of dialysis exchanges, medication, diet/fluid or exercise; did not report on methods to measure or define (non-) adherence; or did not present numerical data on (non-) adherence for PD patients separately to HD. Studies reporting on performance of different steps of PD protocol procedures such as preparation of materials, sterilization, connection/disconnection, or disposal of dialysate bags rather than performance of dialysis exchanges per se were also excluded. These were deemed more related to quality of performance of the recommended procedures among patients who perform PD exchanges rather than (non-) adherence to dialysis exchanges.

### Data extraction

Data from the studies were extracted by one analyst (HL) and second analyst (KG) verified all extractions against the original studies. Information extracted included: authors, year of publication, country, study design, PD modality, age, gender, adherence assessment method, definition of non-adherence, non-adherence rates, and factors associated with adherence/non-adherence. Where information on mean age and proportion of male vs. female study participants were unavailable, estimates were calculated based on available data.

We grouped non-adherence into three categories: non-adherence to dialysis exchanges (e.g., missing, shortening or altering schedules), medications (which include not only prescribed phosphate binders but also other medications, e.g., erythropoietin), and dietary/fluid restrictions. When studies did not distinguish between non-adherence rates of their modality sub-population (APD/CAPD), we extracted the available data for the overall study sample.

### Quality assessment

The methodological quality of the studies was assessed using a shortened version of the Effective Public Health Practice Project (EPHPP) Quality Assessment Tool for Quantitative Studies [25] employing only the sections pertaining to selection bias, data collection and withdrawals/dropouts. Other sections were not used as they were tailored towards interventional, comparative study designs and were not deemed relevant to many of the studies included in this review. Two researchers (KG and AYL) assessed the quality of the studies independently. Discrepancies were resolved by discussion until consensus was reached.

## Results

### Search results and study characteristics

**Figure 1:**
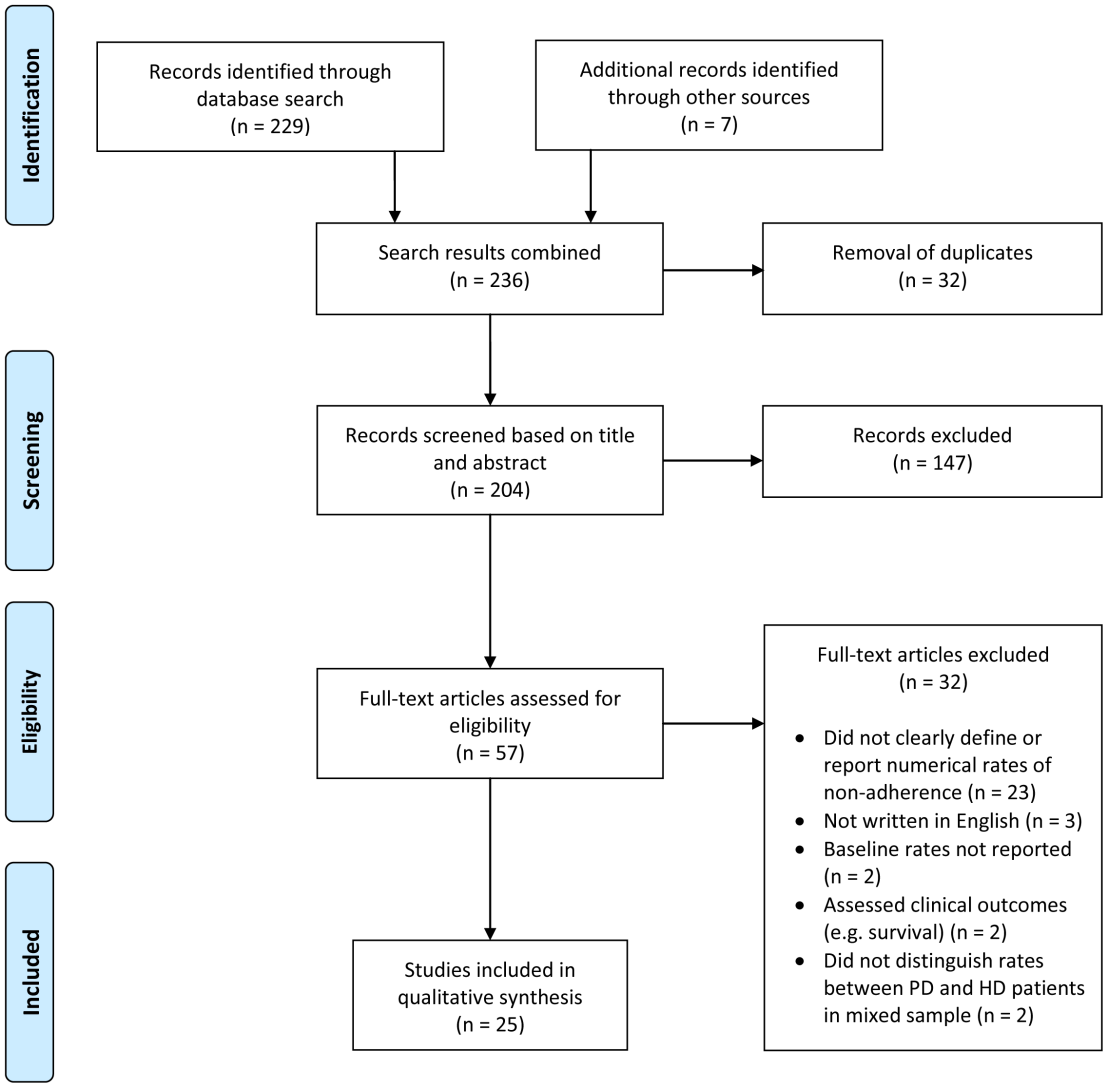
Flowchart of Study Selection.

Figure 1 shows the flow of literature into this systematic review. We obtained a total of 204 articles from electronic databases and additional searches, of which 147 were excluded at the title/abstract screen stage. Full papers and references lists were reviewed for the remaining 57 studies. A final total of 25 (out of the identified 204) studies were judged to meet the criteria for inclusion in the review (see Table 2). The main reasons for exclusion at both the abstract and full paper screens were that studies: did not clearly define or report the numerical rates of non-adherence, were not written in English, assessed the effects of biochemical markers on clinical outcomes such as survival, were intervention studies aimed at improving adherence without baseline rates reported, or did not distinguish between adherence rates in HD and PD patients among the mixed sample pools.

**Table 2:** Non-Adherence Rates Documented in PD Patients.

Author, Pub. Year	Area	Sample		Design	Method	Definition of NA	NA Rates		
		Modality	Mean Age (% Female)^a^				Dialysis	Medication	Diet
Amici et al., 1996	Italy	50 PD	65.9(–)	Cross-sectional	Self report	Not always observing dialysis prescription (time frame not stated)	18%	–	–
					PD Adequest Software	The % of patients below 95% CI of mean difference between measured and estimated weekly CrCl derived as per software in the patients reported no deviations	22%		
Bernardini et al., 1997	USA	15 CAPD; 5CCPD	46.8 (45)	Cross-sectional	Inventory check	Performing less than 90% of prescribed exchanges over interval period of 4–8 weeks	40%	–	–
Bernardini et al., 1998	USA	35 CAPD; 15 CCPD	48.7 (50)	Cross-sectional	Inventory check	Performing less than 90% of prescribed exchanges over interval period of 1–3 months	35%	–	–
Bernardini et al., 2000	USA	92 PD	52.8 (22)	Cross-sectional	Inventory check	Performing less than 90% of prescribed exchanges over interval period of 6 months, or until transplantation for 4 years	30%	–	–
Blake et al., 2000	USA	656 PD	56.0 (52)	Cross-sectional	Self-report	Missing ≥1 exchange per week, or ≥2 exchanges per month	15%	–	–
Chan et al., 2009^b^	Hong Kong	76 CAPD	66.1 (60.5)	Cross-sectional	Self-report (DDFQ)	Mild, moderate, severe, or very severe deviation from therapeutic regimen (i.e., >0)	2.6%	3.9%	Diet/Fluid: 34.2/55.9%
		77 CAPD	53.1 (31.2)				39%	37.7%	14.4%/33.8%
Chen et al., 2006	China	35 PD	57.6 (57)	Prospective	Laboratory data	Excessive dietary protein intake (DPI) beyond the range 0.8–1.2 g/kg/d	–	–	67%
Chua et al., 2011	USA	51 APD	11.8 (43)	Retrospective	Baxter Home-Choice PRO Card	Following a prescription variable (number of sessions/duration of cycles/number of cycles/dialysate volume) by ≤95% of the time	18%/4%/8%/22%	–	–
						Following a prescription variable (number of sessions/duration of cycles/number of cycles/dialysate volume) by <90% of the time	12%/6%/2%/2%		
Figueiredo et al., 2005	Brazil	30 PD	52.8 (63)	Cross-sectional	Self-report	Performing less than 90% of prescribed exchanges over interval period of 1–2 months for 4 months	30%	–	–
Fine, 1997^b^	Canada	67 CAPD	–	Cross sectional	Records of dialysate delivery and pick-up visit)	Using less than 90% of prescribed dialysate based on data (over a mean of 18 months)	3%	–	–
		77 CAPD		Retrospective	Records of dialysate delivery and pick-up visit)	Using less than 90% of prescribed dialysate based on data (over a mean of 18 months in retrospective)	5%		
		26 CAPD		Prospective	Inventory check (nurse)	Using less than 90% for an interval period of 4–6 weeks	12%		
García-Llana et al., 2013	Spain	31 PD	47.9 (19)	Cross-sectional	Self-report (Morisky-Green-Levine Test)	At least one positive (non-adherent) response on the Green-Levine Test	–	82.8% (antihypertensives); 85.2% (phosphate binders)	–
Hall et al., 2004	USA	2001 PD	53.7 (49)	Prospective	Nurse evaluation	At least 3 of the following: not attending clinics, ordering enough supplies, avoiding expedited delivery, bringing adequacy samples	10%	–	–
Hollis et al., 2006	Belgium, Italy, UK	149 APD 227 CAPD	60.0 (45)	Cross-sectional	Self-report	Any modification of treatment regimen in the past month	20%	–	–
Hung et al., 2013	Taiwan	76 PD	43.6 (62)	Cross-sectional	Interview	Not meeting all of: taking full prescribed dose, taking dose regularly with meals, and taking dose using correct method	–	43% total	–
Juergensen et al., 2004	USA	42 APD	58.0 (43)	Prospective	Baxter Home-Choice PRO Card	(Delivered dialysis volume/prescribed dialysis volume)*100<90%	17%	–	–
Kutner et al., 2002	USA	21 APD; 30 CAPD	49.2 (51)	Cross-sectional	Self-report/Laboratory data (Medication)	*APD*: Missed/shortened at least 1 session during past 2 weeks	30% (missed);10% (shortened APD)	10%	–
						*CAPD*: Missed at least 1 session during past 7 days			
						*Medication*: Serum phosphate >7.5 mg/dl over 3 months			
Lam et al., 2010	Hong Kong	173 CAPD	Median = 60 (49)	Cross-sectional	Self-report (DDFQ)	Mild, moderate, severe, or very severe deviation from therapeutic regimen (i.e., >0)	7%	17%	Diet/Fluid: 62%/36%
Neri et al., 2002	Italy	19 APD	67.9 (21)	Retrospective	Baxter PD Link software	Missing PD sessions in 90 days	53%	–	–
Quan et al., 2006	China	30 PD (diabetic)	65.4 (53)	Prospective	3-day Dietary diary and fluid intake history	Deviating from dietician's instructions	–	–	19.5%
Rivetti et al., 2002	Italy	23 APD	68.0 (25)	Prospective	Baxter Home-Choice PRO Card	Number of missed sessions in 30 days	4.3%	–	–
Russo et al., 2006	Italy	191 PD	61.0 (41)	Prospective	Nurse evaluation (home visit and completion of nurse scoring card)	*Dialysis*: deviation from exchange protocol procedures/excessive of inadequate quantity of supplies present	23%/10%	25%	–
						*Medication*: incongruence between drugs at home and prescribed in the clinical file			
Sevick et al., 1999	USA	20 CAPD	56.2 (42)	Prospective	Self report (2 week log); APREX Medication Event Monitoring System	Missed exchanges (self report/ MEMS)	2.5%/22.7%	–	–
						Missing ≥10% of exchanges	36.8%		
						Missing >50% of exchanges	26%		
Warren et al., 1994	USA	64 PD	53.0 (–)	Cross-sectional	Self report (patient)	Non compliance to prescribed exchanges (time frame not stated)	14%	–	–
					Laboratory data	The % of patients with 24% difference between measured and estimated creatinine (cut-off based on (99% CI of the patients reported either compliance of non-compliance in the history taking)	26%		
Wazny et al., 2002	Canada	54 PD	55.9 (56)	Cross-sectional	Pharmacy record review; Self-report	Using less than 90% of prescribed dose in EPO treatment as per records or reporting missing EOP injections	–	35%	–
Yu et al., 2012	Singapore	15 APD; 5 CAPD	64.4 (40)	Cross-sectional	Self-report; Laboratory data	*Dialysis*: Skipping or shortening exchanges at least 1 exchange during past 4 weeks	15% total (5% APD /10% CAPD)	15%/30%/16%	26%/26%/16%
						*Medication*: Skipping/forgetting doses/Serum phosphate >1.78 mmol/L			
						*Diet*: intentional/unintentional dietary plan deviations / Serum potassium <3.5 mmol/L			

Note: PD  =  Peritoneal Dialysis. HD  =  Haemodialysis. CAPD  =  Continuous Ambulatory PD. APD  =  Automated PD. CCPD  =  Continuous Cycling PD. NA  =  Non-adherence. DDFQ  =  Dialysis Diet and Fluid Questionnaire. EPO  =  Erythropoietin. ^a^Age and gender characteristics are presented only if reported in (or able to be calculated from) the study. ^b^Articles that included more than one patient sub-group, hence sample size was separated to accurately reflect adherence rates reported in these studies.

### Study locations and settings

As seen in Table 2, approximately half (N = 12) of the included adherence-related studies were conducted in North America [3], [4], [23], [26], [27], [28], [29], [30], [31], [32], [33], [34], six were conducted in Europe [35], [36], [37], [38], [39], [40], and six studies were conducted in Asia [41], [42], [43], [44], [45], [46]. One study was conducted in South America [47].

### Patient populations and sample sizes

All of studies included patients on PD yet exact PD modality (i.e. CAPD, APD/Continuous Cycle Peritoneal Dialysis; CCPD) was not always clearly stated (N = 12). Of those where modality was specified, N = 4 studies included CAPD only [29], [32], [41], [44], N = 4 included APD only [28], [31], [38], [39] and N = 5 included both CAPD and APD/CCPD patients [3], [23], [26], [37], [46]. More often than not adherence rates were reported for the pooled PD sample and not separately for the different PD modalities with the exception of adherence to dialysis prescription, where rates for APD and CAPD were separately provided [3], [26], [37], [46].

Sample sizes varied greatly from N = 19 in [38] to N = 2001 in [30]; only 20% (N = 5) of the studies had sample sizes of more than 100 participants [27], [30], [37], [40], [44] (Table 2).

More than half (N = 15) of the included studies were cross-sectional [3], [4], [23], [26], [27], [33], [34], [35], [36], [37], [41], [43], [44], [46], [47], with another seven as longitudinal [30], [31], [32], [39], [40], [42], [45]. Two were retrospective investigations [28], [38]. One study reported presented a mixture of cross sectional/retrospective data with some overlap in their sample [29].

While there was no limit to the dates of identification of studies and the earliest was published in 1994 [33], the majority (N = 19) were conducted and published in or after 2000.

### Study quality

Overall, the studies were judged as being of moderate quality, as measured using the Effective Public Health Practice Project tool. The most common reasons for being of moderate quality were that non-validated tools were used to assess adherence and that the study recruited small numbers of volunteers, or the selection procedures were not outlined so representativeness could not be inferred.

### Definition and measurement of non-adherence

Although some studies report studying adherence and others report non-adherence the studies are implicitly studying both adherence and non-adherence as the one is the converse of the other. Most studies used the same, conceptual definition for non-adherence, namely ‘not following doctor's instructions’. This was operationalized in 20 studies as ‘performing less than prescribed dialysis or deviating from instructions' and in one study as not meeting clinical targets, while four studies used both definitions. Notably, one study [37] chose to avoid the use of term “non-adherence/non compliance” in favor of the term “any modification on PD regime”. Although all studies included some form of definition of non-adherence, the timeframes of measuring non-adherence were not stated in all studies.

The majority of the studies (80%; N = 20) examined adherence with regards to only one aspect of treatment regimen – either dialysis procedures [3], [4], [26], [27], [28], [29], [30], [31], [32], [33], [35], [37], [38], [39], [47], medication [34], [36], [43] or diet/fluid restrictions [42], [45]. Two studies considered at least two of these aspects [23], [40] and another three studies considered all three aspects of treatment (dialysis, medication and diet) [41], [44], [46].

There was a greater degree of research that focused on adherence to dialysis and medication as opposed to dietary recommendations. Adherence to dialysis exchanges was the most commonly assessed treatment aspect (80%; N = 20) [3], [4], [23], [26], [27], [28], [29], [30], [31], [32], [33], [35], [37], [38], [39], [40], [41], [44], [46], [47], eight studies assessed adherence to medication [23], [34], [36], [40], [41], [43], [44], [46] and five studies assessed adherence to diet/fluid restrictions [41], [42], [44], [45], [46]. None of the studies assessed adherence to physical activity despite the fact that it is widely recommended for patients on dialysis and has been found to improve clinical and psychological outcomes.

The methods used to assess non-adherence fell into three categories: (1) subjective measures based on patient self-report, or reporting by nurses/physicians (2) objective/direct measures based on inventory checks/delivery records, built-in software or electronic monitoring systems (e.g. Baxter Home-Choice Pro Card or Baxter PD Link software) and (3) physiological and biochemical indicators that included micronutrients (e.g. serum phosphate, serum potassium), and interdialytic weight gain to evaluate respectively adherence to diet and to fluid intake. Serum creatinine levels were used to quantify adherence to dialysis prescription.

By far, the most frequently used method to assess (non-) adherence across all treatment aspects was self-report either by the patient or healthcare provider (nurse or physician) [3], [4], [23], [26], [27], [32], [33], [34], [35], [36], [37], [40], [41], [43], [44], [45], [46], [47], [48]. Specific methodologies varied across studies. A total of 17 studies used a self-report by interview or an ad hoc (non-validated) patient questionnaire to measure adherence whereas three studies used self-report with a validated questionnaire (Morisky-Green-Levine Test; Dialysis Diet and Fluid Non-adherence Questionnaire) to measure adherence [36], [41], [44].

Biochemical measures/markers were used to quantify adherence to medication (e.g., serum phosphate for phosphate binders [23], [46]) or diet (e.g., protein intake and serum potassium [42], [46]). Only one study used creatinine levels as a marker of adherence to dialysis [33].

Seven out of the 25 reviewed studies used two or more adherence instruments [23], [29], [32], [33], [34], [35], [46], and one [34] combined the different instruments in their analyses to estimate non-adherence rates.

### Occurrence of non-adherence

Overall, non-adherence rates ranged from 2.6% to 85.2%. The lowest non-adherence rates were those that measured adherence with built-in software/dialysis delivery records and the highest non-adherence rates measured adherence by either patient self-report or laboratory data. The rates differed across treatment aspects and various definitions for non-adherence. Estimates were typically higher when non-adherence was defined as any deviation from prescribed or recommended activity, and were lower when more specific criteria were applied (e.g. clinical targets for serum biochemistry or duration or numbers of cycles for APD). These issues are presented below.

### Non-adherence to dialysis procedures

Non adherence to dialysis was measured in 20 of the 25 studies, and was typically defined as missing exchange(s) [27], [28], [32], [38], [39], [41], [46]; shortening sessions [28], [46] (relevant only to APD/CCPD), or using less than the prescribed amount of dialysate, typically verified by delivery records or built-in software in PD cyclers [3], [4], [23], [26], [28], [29], [31], [32], [47] (e.g. Baxter Home-Choice PRO Card or Baxter PD Link Software) (see Table 2). In the study with the largest sample size, non adherence to dialysis encompassed a range of distinct behaviors and was defined as doing at least three of the following: not attending clinics, not ordering enough supplies, avoiding expedited delivery, or not bringing adequate samples to scheduled outpatient PD appointments [30].

Criteria and the window of observations varied. For 40% of the studies (N = 8/20) that investigated adherence to dialysis, performing less than 90% of prescribed exchanges typically over a period of 1 to 6 months was considered as indicative of non-adherence [3], [4], [26], [28], [29], [30], [31], [47]. The remaining studies had varied criteria to identify non-adherence. Four studies (20%; N = 4/20) adopted more inclusive criteria, i.e. any deviation from regime or procedures [37], [40], [41], [44], or at least one missed exchange during either the past one, two or four weeks [23], [27], [46] while others opted for a more rigid approach in which non adherence was signified by missing 50% of more of the exchanges [32]. Indirect methods based on physiological data (biochemistry or dialysis adequacy data) were also employed mainly in earlier studies [33], [35].

Appreciating the heterogeneity of methods, the observed rates of non-adherence to dialysis procedures ranged from 2.6–53%. In general, rates of non-adherence to dialysis exchanges based on self-report ranged from 2.6–39%, while rates based on objective/direct measures such as inventory/delivery records or built-in software in PD cyclers ranged from 3–53% [28], [29], [31], [32], [35], [38], [39].

Missing PD exchanges/sessions was reported to be in range of 2.5–53% [28], [32], [38], [39], [46], shortening (as in reducing duration of cycles or number of cycles) by 4–15% of patients [23], [28], [46] and performing less than 90% of prescribed exchanges (as per dialysate volume) was evident in 2–40% of patients. Indirect biochemical/physiological measures (e.g. creatinine levels and/or analysis urine/dialysate data and peritoneal equilibration tests) indicate non-adherence rates at 22–26% [33], [35].

Over half of the studies (65%; N = 13/20) reported the rates of non-adherence to dialysis procedures to be higher than 20% [3], [4], [23], [26], [28], [32], [33], [35], [37], [38], [40], [41], [47] suggesting that non-adherence to dialysis prescription estimates are closer to upper bounds estimates reaching 25–30% of patients on PD regimes. In the largest study to date (N = 2001) that employed composite indices, a total 10% of PD patients were found to be non-adherent to dialysis exchanges based on the definition of doing at least three of the following: not attending clinics, ordering enough supplies, avoiding expedited delivery, or bringing in their adequacy samples at scheduled outpatient PD appointments [30].

It is important to note that there is no clear consensus on definitions regarding non-adherence to dialysis sessions, which is likely to have attributed to the high degree of variability in reported non-adherence rates. Missing was defined as absence of one or more session per week, or two or more sessions per month [27]. Definitions were also different between CAPD and APD; non-adherence to the former was conceptualized as missing at least one session during the past week, while for the latter as missing at least one session during the past two weeks [23].

### Non-adherence to medication

Non-adherence behaviors to medication included: not completing the full course of a prescribed medication (non-persistence), or incorrectly taking or missing doses of medication.

The range of non-adherence to medication was 3.9–85% (see Table 2 for details). Further investigation revealed that this large spread was due primarily to one outlier, which used more inclusive criteria for non-adherence: i.e., at least one instance of a non-adherent response on the eight-item Morisky-Green-Levine Test (self-report) for anti-hypertensive medication [36]. Removing this paper indicated a range of non-adherence to medication of 3.9–43% for the remaining seven papers.

Both renal specific medications (e.g. phosphate binders, erythropoietin) [23], [34], [36], [43], and other generic/non-renal medications (e.g. medication for extra-renal morbidity such as hypertension) [36] were studied. Several studies assessed non-adherence to medication in general but not to a specific prescribed medication(s) (e.g. [44]). Non adherence rates to generic/non-renal medications ranged from 3.9–37.7% for self-report [41], [44], [46], compared to non-adherence rates of 25% by pill count [40].

Despite the importance of phosphate control in dialysis [49], [50], only three of the studies focused on use of phosphate binders in PD, with self-reported non-adherence rates ranging from 15–85.2% [36], [46] and estimates based on (serum phosphate levels) being more conservative at 10–16% [23], [46].

### Non-adherence to diet/fluid restrictions

Out of all identified studies, only a fraction (20%; N = 5/25) investigated dietary non-adherence in PD patients, with 14.4–67% of patients found to be non-adherent to their dietary guidelines. Of these five, two examined fluid adherence presenting the rate of self-reported non-adherence to fluid restrictions as 33.8–55.9% [41], [44]. Studies that used self report showed non-adherence to diet ranging from 14.4–62% [44], [45]. Unintentional and intentional dietary non-adherence behaviors were equally common at 26% [46]. Biochemical indicators of non-adherence produced divergent findings, with rates of dietary non adherence at 16% based on potassium levels [46] while a total of 67% of patients had excessive dietary protein intake in another study [42].

### Factors associated with non-adherence

To explore factors associated with non-adherence in the primary studies we have adopted a narrative synthesis approach. The focus was on directionality of associations rather than the magnitude as the variation on type of statistical analyses and inconsistent reporting did not allow a more effective synthesis of results. This involved tabulating factors examined in the included studies, their reported relationship with non-adherence outcomes, defined in terms of significance and direction (negative, positive, or no relationship), and tallying studies falling into each respective grouping with the majority of studies falling into any specific category being considered to indicate a likely relationship.

Nine studies (36%; N = 9/25) [23], [27], [28], [34], [36], [37], [41], [44], [46] identified in this review evaluated factors associated with non-adherence in PD. The focus was mainly on socio-demographic parameters (i.e. age, employment status, education level, sex, race, household income and smoking status), followed by medical/treatment-related factors (duration of renal replacement therapy, presence of carer, number of comorbidities, contact with healthcare professionals) [23], [27], [28], [34], [37], [41], [44], [46]. Psychosocial resources variables (i.e. self-efficacy, perceived burden/control, Quality of Life, satisfaction) have not been examined by more than one study per parameter [23], [36]. Five of the studies that explored factors associated with non-adherence relied on univariate and correlations analyses [28], [36], [37], [44], [46], with four using more rigorous multivariate methods [23], [27], [34], [41].

Although some variables were identified to influence non-adherence, overall there was little agreement between the studies on observed associations to allow identification of high risk sub-groups or determinants in terms of predisposing or maintaining factors. Out of the parameters that have been examined by more than one study, consistent associations with non-adherence were identified for five factors: younger age [23], [34], [37], [41], [44], being employed [27], [37], [41], [44], [46], being male [28], [41], [44], being on treatment for a longer period of time [34], [37], [44], and non-white ethnicity [23], [27], [28]. The correlation between education and adherence levels produced mixed results – lower education was shown to be associated with non-adherence in one study [44], but an opposite trend was observed in three other studies [27], [34], [41].

There is limited evidence for psychosocial factors as each of the various parameters were not examined by more than one of the studies included in this review. The patterns of associations however suggest that non-adherence is associated with low self-efficacy [46], high depression and low quality of life [23] or poor satisfaction with treatment [37]. The presence of a caregiver was found in two studies to be associated with lower rates of non-adherence to dialysis [27], [46].

The association of PD modality with adherence outcomes received very little attention. We are unable to provide clear evidence for the role of PD modality as this issue has not been explored in most studies that have recruited both CAPD and APD patients or studies merged patients on PD modalities and reported overall PD non-adherence rates. Based on the limited number of studies to report rates separately for CAPD and APD patients [3], [26], , non-adherence to APD procedures range from 5–20%, in comparison to 10–47% in CAPD. Although no systematic comparisons have been conducted, a trend of higher non-adherence rates in CAPD compared to APD patients is evident.

## Discussion

This is the first systematic review to summarize data on (non-) adherence rates in PD and to identify factors influencing adherence in this patient group. Overall, 25 studies fulfilled the inclusion criteria with the majority focused solely on adherence to dialysis procedures/exchanges or medication, and only five on adherence to diet/fluid. As noted in previous reviews of the adherence literature across a range of patient populations, heterogeneity in methods used is more the rule than the exception. Data source and quality, sample size, and definitions of non-adherence and methods used in the included studies varied widely, thus limiting comparability and summation of results. Methodological variation is expected as there is no ‘gold standard’ to measure adherence [51] nor any clinical ‘benchmark’ on levels of adherence required for clinical benefits in PD. In the studies reported here, cut-offs of performing less than 50%, 90% or 95% of dialysis exchanges were employed as definitions of non-adherence.

Nevertheless, despite disparate operationalizations of non-adherence, evidence indicated that a substantial proportion of patients on PD regimes reported or were found to deviate from prescribed dialytic, medication regimens or dietary recommendations. The overall rates were 2.6% to 85%, with most studies reporting non-adherence rates over 30%. Most notably, regardless of methods used to operationalize non-adherence (i.e., self-report, software, delivery records) non-adherence rates were closer to upper bound estimates than lower bound rates. Non-adherence across the different treatment aspects ranged up to 53% for dialysis procedures, 43% for medication (85% when over-inclusive definition was employed) and 67% for dietary guidelines, confirming that adherence to all key aspects of PD regime is generally poor. In general, although non-adherence rates were somewhat higher for medication and diet compared to dialysis procedures, the rates of missing dialysis exchanges/sessions were far from negligible. In most studies, more than 20% of PD patients performed less than 90% of prescribed exchanges. Given the potential repercussions of non-adherence such as technique failure, peritonitis, and hospitalization [3], [4], these rates are alarming.

Comparing these data in PD to those in HD [2], [10], non-adherence rates to dialysis ranged from 4–53% for PD, as compared to 35% in HD, indicating higher non-adherence in PD patients. Non-adherence to medication and diet on the other hand appears to be lower in PD (3.9–43% and 14.4–67% respectively) relative to those reported in HD, where non-adherence to medication has been reported to range from 3–80.4% [52], [53] and non-adherence to diet between 24–81.4% [54], [55], [56], [57]. The intermittent nature of HD necessitates more rigid dietary requirements relative to PD, which may account for the divergent findings. It is notable however that diet or medication intake (especially related to particular types of medications) have largely been overlooked in the adherence literature in PD, hence making it difficult to assess the true extent of the problem in this population. For instance, only eight studies were identified in our review that looked at adherence to phosphate binders and dietary behaviors in PD. The small sample sizes do cast doubts on the precision of the estimates and their generalizability in PD population, yet the findings from these small studies suggest that a problem of non-adherence does exist in this area. Likewise there have been no studies that examine exercise or other important self-management/self-care behaviors in PD, such as prevention, recognition and help-seeking behaviors in response to signs of infection. Practices and skills related to connect/disconnect, hygiene or sterilization procedures are also important [58]. A study on CAPD patients indicated poor performance of the CAPD steps (most notably not using face mask) for 16.5% patients [59]. Clearly, more research is warranted to explore these behaviours and practices in both CAPD and APD as they may be linked to clinical outcomes such as technique or patient survival.

Based on four studies that compared rates of non-adherence to dialysis prescriptions in APD and CAPD populations in the same study, CAPD patients exhibited higher non-adherence to exchanges (10–47%) than APD (5–20%) patients [3], [26], [37], [46]. This might be related to the procedural differences between the two techniques. CAPD requires multiple manual exchanges administered by the patient/care-giver, whereas APD requires only one overnight session. Higher non-adherence might occur in CAPD given the higher frequency of exchanges leading to greater opportunity to detect non-adherence.

Only one of the included studies explored rates of intentional and unintentional non-adherence [46]. In line with work with other patient populations, unintentional non-adherence was found to occur more frequently. More research will need to be undertaken in this area as these two types of non-adherence lead to very different interventions to improve adherence.

The second goal of this review was to identify factors that are associated with non-adherence in PD. Nine studies identified in this review have investigated potential determinants or correlates with adherence. Considering the lack of adequate statistical power due to small sample size and the suboptimal quality of analyses we would call for caution in the interpretation of associations, and emphasize the need for further work. However, being younger in age, male, employed, of non-white ethnicity or on PD treatment for longer was found to have consistent associations with non-adherence in PD. Patients' self care ability and/or presence of a caregiver may moderate the effect of age [46], [60] as older patients assisted by a caregiver were found to be less likely to miss exchanges than an older patient who is conducting the exchanges him/herself [61].

The role of psychosocial factors of adherence received little attention despite evidence from reviews in other patient populations and HD [10], [62]. Two studies have assessed some factors but none has explicitly used a theoretical framework to understand what facilitates and what inhibits adherence. There is some limited evidence that low Quality of Life, low satisfaction, low self-efficacy and depression are associated with non-adherence but replication is necessary as none of these factors was identified in more than one study. It is only once these associations are conclusively known that evidence-based interventions to increase adherence can be developed and tested.

A first step to improving adherence is being able to define, assess and recognize it. Arriving at a consensual definition for non-adherence in the context of PD and developing reliable methods of assessment so as to establish accurate frequencies of non-adherence are essential to determine the extent of the problem and provide basis for prevention, support and intervention that can improve care and outcomes for PD patients. Although methods are not yet available for routine use, renal health care professionals should regularly enquire of patients as to how they manage their treatment requirements so that difficulties can be identified early and action or support can be rendered.

To our knowledge, this is the first comprehensive systematic review to focus on adherence in PD, and it has been conducted according to PRISMA guidelines. Despite the rigorous methodology used to search, select and extract data, the study has several limitations, most of which are inherent to the studies included. First, evidence gathered is based largely on cross-sectional data. We found that recruited study samples were very small, thus limiting the generalizability of findings. Only five studies had sample sizes above 100 and the majority of studies opted to merge between APD and CAPD groups due to small sample sizes. Longitudinal data are needed to evaluate course of adherence over time in the PD population. Second, there was very little consistency in methodologies and the description and rigor of self-reported measures of adherence were generally poor.

We had also hoped to compare non-adherence across PD modalities but only four studies reported adherence separately for modality subgroups, hence limiting any analysis. Other possible limitations of this study is the potential publication bias introduced by excluding non-English studies and not conducting a search for grey literature via contacting relevant experts for unpublished manuscripts due to limited resources and rapid time frame for the review. Finally our approach to rely on directionality and statistical significance when exploring factors associated with non-adherence, albeit deemed necessary to overcome caveats in the reporting of relevant statistical data failed to consider the magnitude of reported effects and may therefore have resulted in taking a conservative stance in interpreting the evidence.

In conclusion, the results of this review suggest that non-adherence is a persistent concern in PD and needs to be given serious consideration in order to improve outcomes. Additional high quality, adequately powered studies are required to investigate adherence to all aspects of treatment particularly with respect to diet, types of medication, lifestyle recommendations and other self-care behaviors that are critical to PD success. The use of multiple measurement methods would be recommended as a triangulation of methods can help gain better understanding and more reliable estimates of rates or magnitude of non-adherence. Due consideration should be given to identifying factors that influence non-adherence as these remain inadequately addressed. The role of PD modality, psychosocial and interpersonal factors guided by relevant theoretical frameworks can advance understandings of non-adherence and inform interventions for this patient group.

## Supporting Information

Checklist S1 **PRISMA Checklist.**
(DOC)

## References

[1] Jain AK, Blake P, Cordy P, Garg AX (2012) Global trends in rates of peritoneal dialysis. J Am Soc Nephrol 23: 533–544. 22302194 10.1681/ASN.2011060607 PMC3294313

[2] Denhaerynck K, Manhaeve D, Dobbels F, Garzoni D, Nolte C, et al (2007) Prevalence and consequences of nonadherence to hemodialysis regimens. Am J Crit Care 16: 222–235. 17460313

[3] Bernardini J, Piraino B (1998) Compliance in CAPD and CCPD patients as measured by supply inventories during home visits. Am J Kidney Dis 31: 101–107. 9428459 10.1053/ajkd.1998.v31.pm9428459

[4] Bernardini J, Nagy M, Piraino B (2000) Pattern of noncompliance with dialysis exchanges in peritoneal dialysis patients. Am J Kidney Dis 35: 1104–1110. 10845824 10.1016/s0272-6386(00)70047-3

[5] Bender B, Rand C (2004) Medication non-adherence and asthma treatment cost. Current Opinion in Allergy & Clinical Immunology 4: 191–195. 15126940 10.1097/00130832-200406000-00009

[6] Sokol MC, McGuigan KA, Verbrugge RR, Epstein RS (2005) Impact of medication adherence on hospitalization risk and healthcare cost. Medical care 43: 521–530. 15908846 10.1097/01.mlr.0000163641.86870.af

[7] Sunanda K, Fadia S (2008) Medication non-adherence is associate with increased medical health care costs. Digestive diseases and sciences 53: 1020–1024. 17934828 10.1007/s10620-007-9968-0

[8] Denhaerynck K, Dobbels F, Cleemput I, Desmyttere A, Schafer-Keller P, et al (2005) Prevalence, consequences and determinants of nonadherence in adults renal transplant patients: A literature review. Transpl Int 18: 1121–1133. 16162098 10.1111/j.1432-2277.2005.00176.x

[9] Denhaerynck K, Burkhalter F, Schäfer-Keller P, Steiger J, Bock A, et al (2009) Clinical consequences of non adherence to immunosuppresive medication in kidney transplant patients. Transpl Int 22: 441–446. 19144090 10.1111/j.1432-2277.2008.00820.x

[10] Karamanidou C, Clatworthy J, Weinman J, Horne R (2008) A systematic review of the prevalence and determinants of nonadherence to phosphate binrding medication in patients with end-stage renal disease. BMC Nephol 9. 10.1186/1471-2369-9-2 PMC2270809 18237373

[11] Khalil AA, Frazier SK (2010) Depressive symptoms and dietary nonadherence in patients with end-stage renal disease receiving hemodialysis: a review of quantitative evidence. Issues Ment Health Nurs 31: 324–330. 20394478 10.3109/01612840903384008

[12] Schmid H, Hartmann B, Schiffl H (2009) Adherence to prescribed oral medication in adult patients undergoing chronic hemodialysis: a critical review of the literature. Eur J Med Res 14: 185–190. 19541573 10.1186/2047-783X-14-5-185 PMC3351975

[13] Lehane E, McCarthy G (2007) Intentional and unintentional medication non-adherence: a comprehensive framework for clinical research and practice? A discussion paper. Int J Nurs Stud 44. 10.1016/j.ijnurstu.2006.07.010 16973166

[14] Bland RJ, Cottrell RR, Guyler LR (2008) Medicaton compliance of hemodialysis patients and factors contributing to non-compliance. Dial Transpl 37: 174–178.

[15] Sabate E (2003) Adherence to Long-Term Therapies: Evidence for Action. Geneva: World Health Organization.

[16] DiMatteo MR (2004) Variations in patients' adherence to medical recommendations: a quantitative review of 50 years of research. Med Care 42: 200–209. 15076819 10.1097/01.mlr.0000114908.90348.f9

[17] Hailey BJ, Moss SB (2000) Compliance behaviour in patients undergoing haemodialysis: A review of the literature. Psychol Health Med 5: 395–406.

[18] Lulowsky L, Mehrotra R, Kheifets L, Arah OA, Nissenson AR, et al. (2013) Comparing mortality of peritoneal and hemodialysis patients in the first 2 years of dialysis therapy: a marginal structural model analysis. Clin J Am Soc Nephrol 8. 10.2215/CJN.04810512 PMC3613949 23307879

[19] Pruthi R, Steenkamp R, Feest TG (2013) UK Renal Registry 16th Annual Report: Chapter 8 Survival and cause of death of UK adult patients on renal replacement therapy in 2012: national and centre-specific analyses. Bristol: The Renal Association. 10.1159/000360027 24662172

[20] Termorshuizen F, Korevaar JC, Dekker FW, Van Manen JG, Boeschoten EW, et al (2003) Hemodialysis and peritoneal dialysis: comparison of adjusted mortality rates according to the duration of dialysis: analysis of the Netherlands Cooperative Study on the Adequacy of Dialysis 2. J Am Soc Nephrol 14: 2851–2860. 14569095 10.1097/01.asn.0000091585.45723.9e

[21] Vonesh E, Snyder JJ, Foley R, Collins A (2004) The differential impact of risk factors on mortality in hemodialysis and peritoneal dialysis. Kidney Int 66. 10.1111/j.1523-1755.2004.66028.x 15569331

[22] Wright M, Woodrow G, O'Brien S, King N, Dye L, et al (2004) Polydipsia: a feature of peritoneal dialysis. Nephrol Dial Transpl 19: 1581–1586. 10.1093/ndt/gfh227 15069171

[23] Kutner NG, Zhang R, McClellan WM, Cole SA (2002) Psychosocial predictors of non-compliance in haemodialysis and peritoneal dialysis patients. Nephrol Dial Transpl 17: 93v99. 10.1093/ndt/17.1.93 11773470

[24] Liberati A, Altman DG, Tetzlaff J, Mulrow C, Gøtzsche PC, et al (2009) The PRISMA statement for reporting for systematic reviews and meta-analyses of studies that evaluate health care interventions: explanation and elaboration. Ann Intern Med 151: W65–94. 19622512 10.7326/0003-4819-151-4-200908180-00136

[25] Effective Public Health Practice Project (1998) Quality Assessment Tool for Quantitative Studies. In: Project EPHP, editor.

[26] Bernardini J, Piraino B (1997) Measuring compliance with prescribed exchanges in CAPD and CCPD patients. Perit Dial Dial 17: 338v342. 9284459

[27] Blake PG, Korbet SM, Blake R, Bargman JM, Burkart JM, et al (2000) A multicenter study of noncompliance with continuous ambulatory peritoneal dialysis exchanges in US and Canadian patients. Am J Kidney Dis 35: 506–514. 10692278 10.1016/s0272-6386(00)70205-8

[28] Chua AN, Warady BA (2011) Adherence of pediatric patients to automated peritoneal dialysis. Pediatr Nephrol 26: 789–793. 21350797 10.1007/s00467-011-1792-2

[29] Fine A (1997) Compliance with CAPD prescription is good. Perit Dial Dial 17: 343–346. 9284460

[30] Hall G, Bogan A, Dreis S, Duffy A, Greene A, et al. (2004) New directions in peritoneal dialysis patient training. Nephrol Nurs J 31: 149–154, 159–163. 15114797

[31] Juergensen PH, Gorban-Brennan N, Finkelstein FO (2004) Compliance with the dialysis regimen in chronic peritoneal dialysis patients: Utility of the pro card and impact of patient education. Adv Perit Dial 20: 90–92. 15384803

[32] Sevick MA, Levine D, Burkart JM, Rocco M, Keith J, et al (1999) Measurement of continuous ambulatory peritoneal dialysis prescription adherence using a novel approach. Perit Dial Dial 19: 23–30. 10201337

[33] Warren PJ, Brandes JC (1994) Compliance with the peritoneal dialysis prescription is poor. J Am Soc Nephrol 4: 1627–1629. 8025237 10.1681/ASN.V481627

[34] Wazny LD, Stojimirovic BB, Heidenheim P, Blake PG (2002) Factors influencing erythropoietin compliance in peritoneal dialysis patients. Am J Kidney Dis 40: 623–628. 12200815 10.1053/ajkd.2002.34925

[35] Amici G, Viglino G, Virga G, Gandolfo G, Ra Rin G, et al (1996) Compliance study in peritoneal dialysis using PD Adequent software. Perit Dial Dial 16: S176–178. 8728188

[36] García-Llana H, Remor E, Selgar R (2013) Adherence to treatment, emotional state and quality of life in patients with end-stage renal disease undergoing dialysis. Psicothema 25: 79–86. 23336548 10.7334/psicothema2012.96

[37] Hollis J, Harman W, Goovearts T, Paris V, Chivers G, et al (2006) Managing peritoneal dialysis (PD) —factors that influence patients' modification of their recommended dialysis regimen. A European study of 376 patients. J Ren Care 32: 202–207. 17345979 10.1111/j.1755-6686.2006.tb00023.x

[38] Neri L, Viglino G, Cappelletti A, Gandolfo G, Barbieri S (2002) Compliance in automated peritoneal dialysis. Adv Perit Dial 18. 12402591

[39] Rivetti M, Battú S, Barrile P, Benotto S, Berruto L, et al. (2002) Compliance with automated peritoneal dialysis. EDTNA ERCA J 28: 40–43, 55. 10.1111/j.1755-6686.2002.tb00197.x 12035903

[40] Russo R, Manili L, Tiraboschi G, Amar K, De Luca M, et al. (2006) Patient re-training in peritoneal dialysis: why and when it is needed. Kidney Int Suppl 70: S127–132. 10.1038/sj.ki.5001929 17080104

[41] Chan MF, Wong FKY, Chow SKY (2009) Investigating the health profile of patients with end-stage renal failure receiving peritoneal dialysis: a cluster analysis. J Clin Nurs 19: 649–657. 10.1111/j.1365-2702.2009.03103.x 20500306

[42] Chen W, Lu X, Wang T (2006) Menu suggestion: An effective way to improve dietary compliance in peritoneal dialysis patients. J Renal Nutr 16: 132–136. 10.1053/j.jrn.2006.01.009 16567269

[43] Hung KY, Liao SC, Chen TH, Chao MC, Chen JB (2013) Adherence to phosphate binder therapy is the primary determinant of hyperphosphatemia incidence in patients receiving peritoneal dialysis. Ther Apher Dial 17: 72–77. 23379497 10.1111/j.1744-9987.2012.01098.x

[44] Lam LW, Twinn SF, Chan SWC (2010) Self-reported adherence to a therapeutic regimen among patients undergoing continuous ambulatory peritoneal dialysis. J Adv Nurs 66: 763–773. 20423364 10.1111/j.1365-2648.2009.05235.x

[45] Quan L, Xu Y, Luo SP, Wang L, LeBlanc D, et al (2006) Negotiated care improves fluid status in diabetic peritoneal dialysis patients. Perit Dial Dial 26: 95–100. 16538882

[46] Yu ZL, Yeoh LY, Seow YY, Luo XC, Griva K (2012) Evaluation of adherence and depression among patients on peritoneal dialysis. Singapore Med J 53: 474–480. 22815017

[47] Figueiredo AE, Santos KS, Creutzberg M (2005) Compliance in peritoneal dialysis measured by supply inventories. Adv Perit Dial 21: 77–79. 16686290

[48] Fine A (1997) Compliance with CAPD prescription is good. Perit Dial Dial 17: 343–346. 9284460

[49] Weed-Collins M, Hogan R (1989) Knowledge and health beliefs regarding phosphate-binding medication in predicting compliance. ANNA J 16: 278–282. 2742396

[50] National Kidney Foundation (2012) Dialysis.

[51] Morris LS, Schulz RM (1992) Patient compliance: an overview. J Clin Pharm Ther 17: 183–195. 10.1111/j.1365-2710.1992.tb01306.x 1464632

[52] Hirth RA, Greer SL, Albert JM, Young EW, John D (2008) Out-of-pocket spending and medication adherence among dialysis patients in twelve countries. Health affairs 27: 89–102. 18180483 10.1377/hlthaff.27.1.89

[53] Lindberg M, Lindberg P, Wikstrom B (2007) Medication discrepancy: A concordance problem between dialysis patients and caregivers. Scandinavian journal of urology and nephrology 41: 546–552. 17853014 10.1080/00365590701421363

[54] Kara B, Caglar K, Kilic S (2007) Nonadherence with diet and fluid restrictions and perceived social support in patients receiving hemodialysis. J Nurs Scholarship 39: 243–248. 10.1111/j.1547-5069.2007.00175.x 17760797

[55] Kugler C, Maeding I, Russell CL (2011) Non-adherence in patients on chronic hemodialysis: an international comparison study. J Nephrol 24: 366–375. 20954134 10.5301/JN.2010.5823

[56] Lin CC, Liang CC (1997) The relationship between health locus of control and compliance of hemodialysis patients. Kaohsiung J Med Sci 13: 243–254. 9177086

[57] Vlaminck H, Maes B, Jacobs A, Reyntjens S, Evers G (2001) The dialysis diet and fluid non-adherence questionnaire: validity testing of a self-report instrument for clinical practice. J Clin Nurs 10: 707–715. 11822521 10.1046/j.1365-2702.2001.00537.x

[58] Bender FH, Bernardini J, Piraino B (2006) Prevention of infectious complications in peritoneal dialysis: best demonstrated practices. Kidney Int 70: S44–54. 10.1038/sj.ki.5001915 17080111

[59] Mawar S, Gupta S, Mahajan S (2012) Non-compliance to he continuous ambulatory peritoneal dialysis procedure increases the risk of peritonitis. Int Urol Nephrol 44: 1243–1249. 22102137 10.1007/s11255-011-0079-7

[60] Blake PG (1999) Individualized prescription of peritoneal dialysis therapy? Perit Dial Dial 19: S495–498. 10406571

[61] Ricka R, Vanrenterghem Y, Evers GCM (2002) Adequate self-care of dialysed patients: a review of the literature. Int J Nurs Stud 39: 329–339. 11864656 10.1016/s0020-7489(01)00024-4

[62] Bane C, Hughes CM, McElnay JC (2006) The impact of depressive symptoms and psychosocial factors on medication adherence in cardiovascular disease. Patient Educ Couns 60: 187–193. 16253468 10.1016/j.pec.2005.01.003

